# Determination of HIV-1 coreceptor tropism using proviral DNA in women before and after viral suppression

**DOI:** 10.1186/s12981-015-0055-x

**Published:** 2015-04-18

**Authors:** Russell E Baumann, Amy A Rogers, Hasnah B Hamdan, Harold Burger, Barbara Weiser, Wei Gao, Kathryn Anastos, Mary Young, William A Meyer, Rick L Pesano, Ron M Kagan

**Affiliations:** Focus Diagnostics, a Subsidary of Quest Diagnostics, San Juan Capistrano, CA USA; University of California, Davis School of Medicine, and Sacramento VA Medical Center, Sacramento, CA USA; Albert Einstein College of Medicine, and Montefiore Medical Center, Bronx, NY USA; Georgetown University, Washington, DC USA; Quest Diagnostics, Baltimore, MD USA

**Keywords:** HIV, Tropism, pvDNA

## Abstract

**Background:**

An HIV-1 tropism test is recommended prior to CCR5 antagonist administration to exclude patients harboring non-R5 virus from treatment with this class of antiretrovirals. HIV-1 tropism determination based on proviral DNA (pvDNA) may be useful in individuals with plasma viral RNA suppression. We developed a genotypic tropism assay for pvDNA and assessed its performance in a retrospective analysis of samples collected longitudinally.

**Results:**

We randomly selected paired plasma/PBMC samples from the Women’s Interagency HIV Study with plasma viral load ≥5,000 cp/mL at time 1 (T1), undetectable viral load maintained for ≥1 year and CD4+ >200 cells/μL at time 2 (T2). pvDNA was isolated from cryopreserved PBMCs. Sequences were analyzed in triplicate from 49/50 women, with tropism assigned using the geno2pheno (g2p) algorithm. The median time between T1 and T2 was 4.1 years. CXCR4-using virus was detected in 24% of the RNA samples and 33% of the pvDNA samples at T1, compared to 37% of the pvDNA samples at T2. Concordance between plasma RNA and pvDNA tropism was 88% at T1 and 80% at T2. The g2p scores for RNA (T1) vs DNA (T1, T2) were strongly correlated (Spearman rho: 0.85 (T1); 0.78 (T2)). In women with evidence of tropism switch at T2 (either R5 to non-R5 or non-R5 to R5), there was a correlation between change in tropism and time. Mean pvDNA viral load decreased by 0.4 log10 copies/106 cells between T1 and T2 (p < 0.0001), but this decrease was not significantly associated with tropism status.

**Conclusions:**

We demonstrated that pvDNA tropism in women with HIV-1 suppression is concordant with prior RNA tropism results, even after a median time of >4 years. pvDNA tropism testing may be useful to determine eligibility of patients with viral suppression to switch to a CCR5-antagonist based regimen as well as for research purposes.

**Electronic supplementary material:**

The online version of this article (doi:10.1186/s12981-015-0055-x) contains supplementary material, which is available to authorized users.

## Background

Human immunodeficiency virus type 1 (HIV-1) infects cells through interaction with the CD4 receptor and one of two coreceptors, CCR5 or CXCR4 [1-3]. CCR5 coreceptor-using virus (R5 virus) predominates in 80-90% of recently infected and treatment-naïve HIV-1 patients, while mixed populations of R5 virus and CXCR4 coreceptor-using virus (non-R5 or “X4” virus) are found in up to 50% of late-stage and antiretroviral treatment (ART)-experienced patients [4-10]. The presence of non-R5 virus is associated with lower CD4+ T-cell counts, higher plasma viral loads, and more rapid progression to AIDS [6,9,11,12]. Small-molecule CCR5 antagonists such as maraviroc can effectively inhibit the interaction of R5 HIV-1 with the CCR5 coreceptor [3,13]. An HIV-1 coreceptor tropism test is required prior to maraviroc administration, to exclude patients harboring non-R5 virus from treatment with this drug (Maraviroc prescribing information, https://www.gsksource.com/gskprm/htdocs/documents/SELZENTRY-PI-MG.PDF).

Many HIV-1-infected individuals undergoing ART achieve undetectable or low levels plasma HIV-1 RNA. Because most plasma RNA tropism tests require at least 1,000 copies/mL of HIV-1 RNA to be present in order to obtain a reportable result, a different type of assay would be required to determine whether individuals with viral suppression could benefit from the inclusion of a CCR5 antagonist in their ART regimen. In this situation, tropism testing of archived HIV-1 proviral DNA (pvDNA) in peripheral blood mononuclear cells (PBMC) can be performed [14,15]. From a clinical standpoint, the ability to determine tropism from pvDNA allows testing to be extended to persons who are taking effective antiretroviral therapy but who may be candidates for CCR5 antagonist therapy. This includes patients who are experiencing adverse effects from their current regimen and patients receiving complex regimens who may benefit from regimen simplification. A number of studies have shown strong agreement between plasma RNA and pvDNA tropism [16-20]. However, few studies have examined pvDNA tropism longitudinally in individuals taking suppressive ART [21-23], and most previous studies of HIV-1 tropism have focused mostly on men [4,8,12,16].

We have developed a genotypic tropism assay that uses triplicate PCR amplification of the HIV-1 envelope V3 region, the major determinant of coreceptor tropism [1,3], and Sanger sequencing of pvDNA isolated from PBMCs [16]. Here we assessed its performance in a retrospective longitudinal analysis of samples from individuals in the Women’s Interagency HIV Study (WIHS), an ongoing long-term observational cohort study of 3,772 HIV-1 infected or at- risk women [24].

## Results

### Baseline characteristics

HIV-1 envelope V3 loop sequences from plasma RNA and pvDNA at T1 and pvDNA at T2 were successfully obtained from 49/50 HIV-positive women and 0/10 HIV-negative control samples. The median interval between time point T1 and T2 samples was 4.1 years (IQR: 2.6, 5.1 years). Baseline characteristics of the subjects are shown in Table 1. There was no statistically significant association between age, viral load at time point T1, nadir CD4+ T-cell count, T-cell counts at either time point or the interval between time points and the tropism concordance status between pvDNA and plasma RNA tropism tests (Table 1). Women with concordant non-R5 pvDNA and plasma RNA tropism results appeared to have a lower nadir CD4+ count (138 cell/uL vs 194 cells/uL overall) but this difference was not statistically significant.

**Table 1:** **Subject Demographic, Virologic and Immunologic Parameters**

		**Coreceptor Tropism Status** ^**2**^			
**Median (IQR)** ^**1**^	**All Subjects**	**Concordant R5**	**Concordant** **non-R5**	**Discordant**	**p-value** ^**3**^
N	49	28	10	11	
Age (years)	38 (33, 43)	39 (32,33)	36 (32, 38)	40 (34, 44)	0.62
Nadir CD4+ (cell/uL)	191 (87, 269)	194 (106, 270)	138 (44, 249)	191 (83, 285)	0.58
T1 CD4+ (cells/uL)	323 (243, 417)	321 (235, 404)	276 (234, 375)	345 (276, 537)	0.70
T1 viral load (log_10_ copies/mL)	4.8 (4.4, 5.3)	4.7 (4.2, 5.3)	5.1 (4.5, 5.4)	4.8 (4.4, 5.3)	0.41
Delta T1 and T2 (years)	4.1 (2.6, 5.1)	3.1 (2.3, 4.7)	4.2 (2.7, 5.4)	4.3 (3.3, 6.2)	0.28
T2 CD4+ (cells/uL)	558 (365, 717)	576 (258, 747)	478 (294, 772)	532 (371, 666)	0.81

^1^T1: Time point 1, viremic subjects pre-suppression; T2: Time point 2, after subjects had undetectable viral load (<80 copies/mL) for ≥1 year.

^2^Concordant R5: CCR5-tropic viral RNA at T1 and proviral DNA at T1 and T2; Concordant non-R5: CXCR4-tropic viral RNA at T1 and proviral DNA at T1 and T2; Discordant: at least 1 discordant tropism result at T1 or T2.

^3^Kruskall-Wallis test for difference in distributions among tropism status categories.

### Coreceptor tropism concordance between plasma RNA and pvDNA

**Figure 1:**
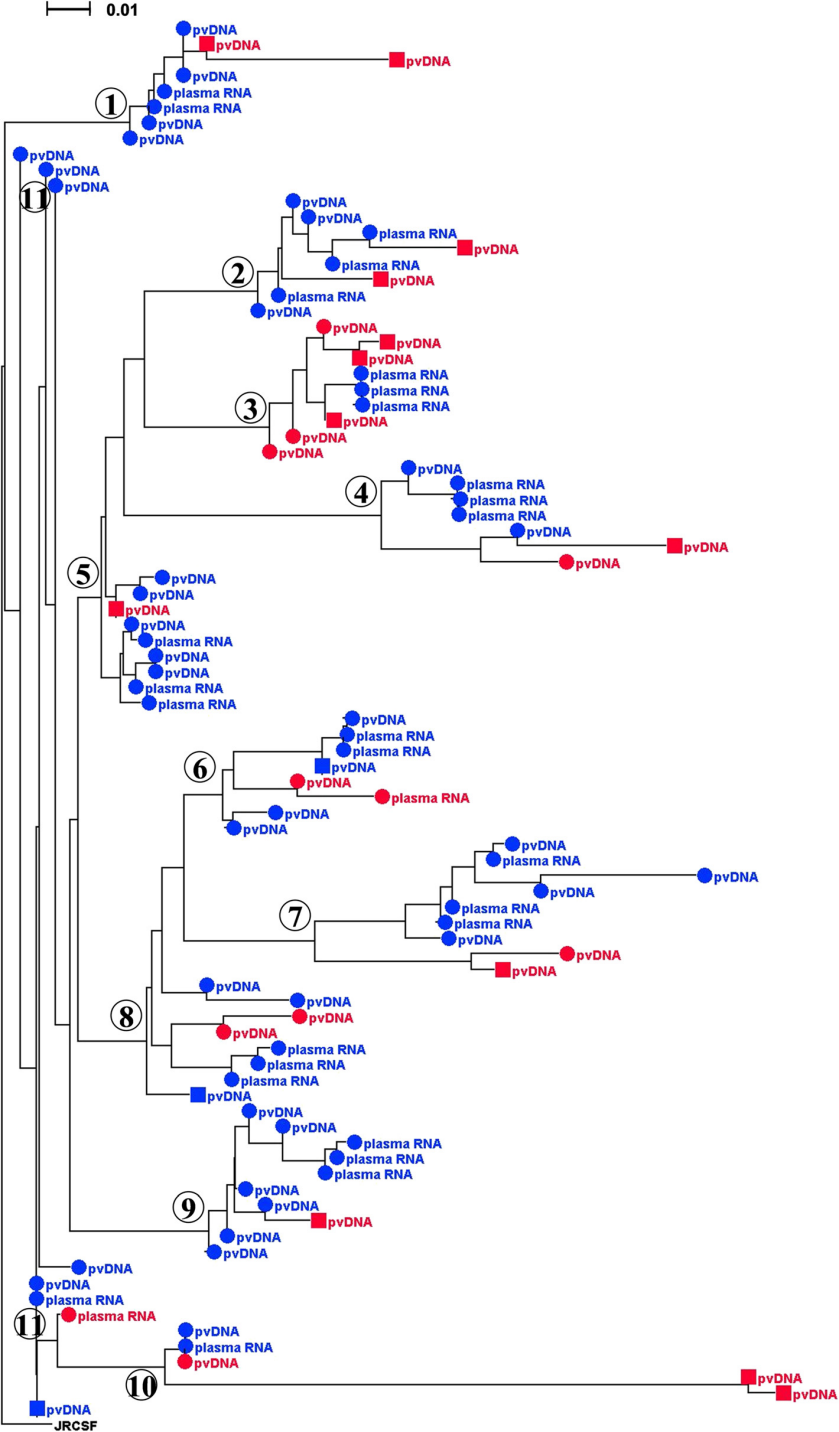
Phylogeny of V3 sequences for discordant tropism group. Blue circles: T1 R5. Red circles: T1 non-R5. Blue squares: TP2 R5. Red squares: T2 non-R5. JRCSF: V3 loop of R5 reference sequence used as an outgroup. Subjects are consecutively numbered 1 – 11. The scale bar is shown in the upper left corner. V3 sequences were aligned with ClustalX version 2.1 (http://www.clustal.org) and a neighbor-joining tree was generated using Kimura-corrected distances for multiple substitutions. The tree was then rendered and illustrated with Denodroscope version 3.2.10 (http://ab.inf.uni-tuebingen.de/software/dendroscope/).

V3 sequences were classified as R5 for 57% (28/49) and non-R5 for 20% (10/49) of the women for analyses of both RNA (T1) and pvDNA (T1 and T2). Tropism results were discordant for 11 women (Figure 1). Four women had only R5 virus detectable in the plasma RNA sample and pvDNA sample collected at T1 but detectable non-R5 virus in the later pvDNA sample from T2 (subjects 1, 2, 5 and 9). An additional four women had only R5 virus in the plasma RNA sample collected at T1 and detectable non-R5 virus for both pvDNA time points (subjects 3, 4, 7, 10). Two women had detectable non-R5 virus in both the plasma RNA sample and the pvDNA sample at T1 but only R5 virus was detected in the pvDNA sample at time point T2 (6 and 11). The last discordant sample had only detectable R5 virus in the plasma RNA sample at time point T1, non-R5 virus in the T1 pvDNA sample, but only R5 virus in the T2 pvDNA sample (subject 8). Phylogenetic tree analysis of the 11 discordant samples showed that the T2 pvDNA sequences remained clustered with sequences from T1 from the same subject, illustrating that they were closely related. The discordant non-R5 viruses tended to appear on distinct branchings within each cluster (Figure 1) showing that they were distinct yet related phylogenetically. None of the discordant sequences clustered with sequences from an unrelated patient indicating that inter-sample contamination is unlikely to account for the observed discordances.

A higher proportion of pvDNA samples were determined to have non-R5 tropism (33% at T1 and 37% at T2) as compared to plasma RNA (24% at T1). The overall concordance of tropism predictions between viral RNA and pvDNA, however, was 88% (kappa = 0.70) at T1; concordance was 80% (kappa = 0.53) at T2 (Table 2). pvDNA was 92% sensitive for non-R5 virus relative to plasma RNA at T1 but the sensitivity was lower at T2 (83%) after a median interval of 4.1 years (Table 2). The geno2pheno FPR scores for coreceptor tropism prediction were highly correlated between RNA and pvDNA at T1 (Spearman correlation coefficient rho = 0.85) and T2 (rho = 0.78), and between the pvDNA results at T1 and T2 (rho = 0.85).

**Table 2:** **Concordance Between Plasma RNA and pvDNA Coreceptor Tropism**

**Test Method**	**Reference Method**	**non-R5 (%) Test Ref.**		**Concord. (%)**	**Sens. (%)** ^**1**^	**Spec. (%)** ^**2**^	**Kappa** ^**3**^
pvDNA (T1)	Plasma RNA (T1)	33%	24%	88%	92%	86%	0.70
pvDNA (T2)	Plasma RNA (T1)	37%	24%	80%	83%	78%	0.53

^1^Sensitivity is defined as concordant non-R5s between the test and reference method/reference method non-R5s.

^2^Specificity is defined as concordant R5s between the test and reference methods/reference method R5s.

^3^Kappa: a measure of inter-rater agreement; 0.41-0.60: moderate agreement; 0.61-0.80: good agreement.

### Lymphocyte counts and pvDNA copy number at T1 and T2

Although CD4+ cell counts did not differ significantly as a function of tropism status, women with non-R5 virus tended to have a lower CD4+ count (Table 1). Women who had only R5 virus at both time points had the largest gain in CD4+ cells (237 cells/μL) over that time period, whereas women harboring non-R5 virus at both time points had a smaller CD4+ cell count increase between T1 and T2 (104 cells/μL). The women with discordant tropism results (defined as having a tropism discordance between either PBMC time point or plasma at T1 and one of the PBMC timepoints), had a median increase of 169 cells/μL. For women with any non-R5 result at either time point, the median increase was 159 cells/μL (IQR: −4.0, +321; p = 0.049). The difference in CD4+ change between the R5 and non-R5 groups was not statistically significant (p = 0.13; Additional file 1: Figure S1).

pvDNA copy number was also measured at both time points. The mean pvDNA viral load decreased from 3.2 log_10_ copies/10^6^ cells at T1 to 2.8 log_10_ copies/10^6^ cells at T2, a significant decline of 0.4 log_10_ copies/10^6^ cells (p < 0.0001; Additional file 2: Figure S2). Changes in proviral DNA viral load were not significantly associated with baseline tropism status.

## Discussion

Investigation of viral evolution during suppressive ART has not documented viral replication in an examination of plasma [25]. Our study compared proviral V3 sequences from PBMCs before and years after viral suppression. There was no evidence of a tropism switch for 38 women with HIV-1 suppression over a median of over 4 years. This observation is consistent with low rates of viral evolution of the V3 loop. Five women with viral suppression had evidence of a tropism switch defined as discordance between the pvDNA tropism results at the two time points, irrespective of the RNA tropism at T1. A potential explanation for this phenomenon could be low level viral replication that was below the limit of quantitation (80 copies/mL) of the plasma viral load assays used in the WIHS. It is also consistent with some viral replication persisting in cells during viral suppression in plasma [21,26]. Clonal expansion of latently infected cells during suppressive ART has also been described [27]. Persistence of expanded clones is often associated with viral integration in genes controlling cell growth. This phenomenon could potentially alter the proportion of non-R5 virus over prolonged periods of virologic suppression. Two other studies that investigated tropism evolution in suppressed patients found lower rates of tropism switching of 7.1% (N = 128, median suppression 4 years) [28] and 9.5% respectively (N = 42, median suppression 2 years) [21]. These studies however, were performed on more recently supressed patients, used a viral load cutoff of 50 copies/mL and included both male and female subjects.

The sensitivity of pvDNA tropism determination at T2 with respect to plasma RNA after a median of 4.1 years of virologic suppression was lower compared to that of the pre-suppression sample T1. Next Generation Sequencing (NGS) of HIV-1 plasma RNA has been shown to predict virologic response to maraviroc as well as the enhanced sensitivity Trofile phenotypic assay and with greater sensitivity for minority non-R5 variants than population sequencing [29-31]. However, the accuracy of NGS for tropism prediction using pvDNA was much lower than population sequencing due to lower specificity [32,33]. Therefore, it is unlikely that NGS would have resulted in improved tropism accuracy for the T2 pvDNA samples. The geno2pheno tropism algorithm that has become the standard tropism prediction tool for genotypic tropism tests also has limitations. A study of plasma viral variants obtained from the “Berlin Patient”, the first patient to be cured of HIV through allogenic transplantation, detected a minority non-R5 viral population by ultradeep sequencing and geno2pheno analysis. This population however, was unable to rebound after transplantation, demonstrating an R5 coreceptor dependency [34]. In spite of these limitations, proviral DNA tropism determinations have proven to be predictive of virologic response in both retrospective studies of viremic patients and more recently prospectively, when used to assign virologically suppressed patients to a maraviroc-based or alternative regimen [33,35].

## Conclusions

In summary, we studied HIV-1 tropism in infected women and found good concordance between plasma RNA and pvDNA coreceptor tropism results which persisted over time: 80% of women had a pvDNA tropism result that was concordant with the baseline plasma finding after a median of 4.1 years and at least a year of viral suppression. Detection of non-R5 virus was slightly more frequent in pvDNA than plasma RNA, as has been observed previously [16,20,33]. This finding suggests that a pvDNA tropism test may exclude slightly more individuals from treatment with a CCR5 inhibitor. However, virtually all women with detectable non-R5 virus in plasma had detectable non-R5 virus in the contemporaneous pvDNA sample, suggesting that pvDNA testing may be a promising screening option for individuals with HIV-1 suppression. 63% of the women in this study had a pvDNA tropism result of R5 at the time of virologic suppression and would have been eligible to switch to a maraviroc-based regimen. Genotypic tropism determination from pvDNA is commercially available at a significantly lower cost than phenotypic tropism testing and may thus allow coreceptor tropism assessment to be extended in a cost effective manner to persons who are taking effective ART but who are being considered for CCR5 antagonist therapy. This includes patients who are experiencing adverse effects from their current antiretroviral regimen and patients receiving complex regimens who may benefit from regimen simplification. This assay may also have value as a research tool for examination of proviral HIV DNA in PBMCs.

## Methods

### Patient Selection

Laboratory testing of WIHS participants is performed during biannual visits and includes lymphocyte subset counts, plasma HIV-1 RNA levels and other tests as described [24]. We randomly selected a cohort of 50 paired samples collected between 1994 and 2006 from WIHS participants in the Bronx, NY and Washington, DC study sites, meeting the following criteria: a plasma viral load ≥5,000 cp/mL at timepoint 1 (T1), a subsequently undetectable plasma viral load of <80 copies/mL that had been maintained for ≥1 year, along with a CD4+ T-cell count of >200 cells/μL at time point 2 (T2). Although most recent studies utilize a viral load cutoff of 50 copies/mL to assess virologic response, a viral load cutoff of 80 copies/mL was selected to be consistent with the limit of quantitation for viral load assays available at the time that these study samples were collected. Ten paired plasma and PBMC samples obtained by the WIHS from HIV-negative women were included as negative controls.

### Plasma HIV RNA coreceptor tropism testing

RNA was isolated from archived plasma specimens (MagNAPure LC automated extraction system, Roche Diagnostics Corp) that were stored at ≤ −70°C. Tropism was determined for 3 independent replicates of viral RNA, by using RT PCR and nested PCR amplification of the V3 loop region from the HIV-1 envelope gene, followed by Sanger DNA sequencing as previously described [16,29]. Tropism assignments (R5 or non-R5) were made with the geno2pheno algorithm [36] with a false positive rate (FPR) of 10% (non-R5: ≤10%) the recommended cutoff when triplicate tropism determinations are employed [14].

### Proviral DNA coreceptor tropism testing

Total DNA was extracted from 0.2 mL of cryopreserved PBMCs (MagNA Pure system, Large Volume MagNA Pure LC DNA Isolation Kit, Roche Diagnostics Corp). Three independent replicates were amplified in 2 rounds of PCR using the same primers that were used for RNA tropism testing, followed by Sanger sequencing as previously described [16,29]. Tropism was assigned using the geno2pheno algorithm [36] using the same cutoff for RNA tropism (non-R5: ≤10%).

HIV-1 pvDNA quantitation was performed using a real-time quantitative PCR kit (Human HIV-DNA qPCR Detection kit, GeneMoRe, Modena, Italy) according to the manufacturer's instructions.

## Additional files

Additional file 1: Figure S1. Changes in CD4+ cell counts between time points T1 and T2 (median, IQR, and range). Additional file 2: Figure S2. pvDNA viral load assayed at T1 and T2 (mean and 95% CI [error bars]).
