# Supplementary figures and images for: Determination of HIV-1 coreceptor tropism using proviral DNA in women before and after viral suppression
Source: AIDS Res Ther. 2015 Apr 18;12:11. doi: 10.1186/s12981-015-0055-x (PMC4403710; doi:10.1186/s12981-015-0055-x)

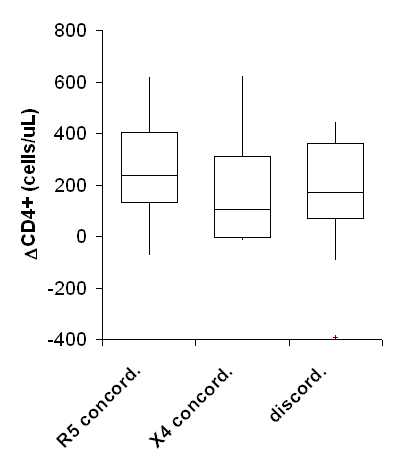

Supplement: Additional file 1: Figure S1. — Changes in CD4+ cell counts between time points T1 and T2 (median, IQR, and range). [file 12981_2015_55_MOESM1_ESM.docx]
