# Supplementary material for: Determination of HIV-1 coreceptor tropism using proviral DNA in women before and after viral suppression
Source: AIDS Res Ther. 2015 Apr 18;12:11. doi: 10.1186/s12981-015-0055-x (PMC4403710; doi:10.1186/s12981-015-0055-x)
Supplement: Supplementary file 2 [file 12981_2015_55_MOESM2_ESM.docx]

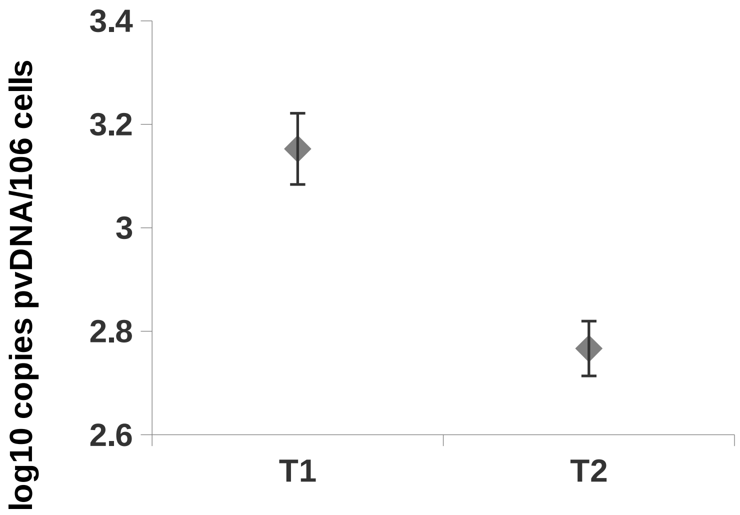

log10 copies pvDNA/106 cells
3.4
3.2
3
2.8
2.6
T1
T2
